# Machine learning in small sample neuroimaging studies: Novel measures for schizophrenia analysis

**DOI:** 10.1002/hbm.26555

**Published:** 2024-03-27

**Authors:** Carmen Jimenez‐Mesa, Javier Ramirez, Zhenghui Yi, Chao Yan, Raymond Chan, Graham K. Murray, Juan Manuel Gorriz, John Suckling

**Affiliations:** ^1^ Department of Signal Theory, Telematics and Communications, Andalusian Research Institute in Data Science and Computational Intelligence (DaSCI) University of Granada Granada Spain; ^2^ Key Laboratory of Psychotic Disorders Shanghai Mental Health Center, Shanghai Jiao Tong University School of Medicine Shanghai China; ^3^ Key Laboratory of Brain Functional Genomics (MOE & STCSM) School of Psychology and Cognitive Science, East China Normal University Shanghai China; ^4^ Neuropsychology and Applied Cognitive Neuroscience Laboratory, CAS Key Laboratory of Mental Health Institute of Psychology, Chinese Academy of Sciences Beijing China; ^5^ Department of Psychiatry University of Cambridge Cambridge UK; ^6^ Cambridgeshire and Peterborough NHS Trust Cambridgeshire UK

**Keywords:** cross‐validation, deep learning, explanaible AI, machine learning, resubstitution with upper bound correction, schizophrenia, sulcal morphology

## Abstract

Novel features derived from imaging and artificial intelligence systems are commonly coupled to construct computer‐aided diagnosis (CAD) systems that are intended as clinical support tools or for investigation of complex biological patterns. This study used sulcal patterns from structural images of the brain as the basis for classifying patients with schizophrenia from unaffected controls. Statistical, machine learning and deep learning techniques were sequentially applied as a demonstration of how a CAD system might be comprehensively evaluated in the absence of prior empirical work or extant literature to guide development, and the availability of only small sample datasets. Sulcal features of the entire cerebral cortex were derived from 58 schizophrenia patients and 56 healthy controls. No similar CAD systems has been reported that uses sulcal features from the entire cortex. We considered all the stages in a CAD system workflow: preprocessing, feature selection and extraction, and classification. The explainable AI techniques Local Interpretable Model‐agnostic Explanations and SHapley Additive exPlanations were applied to detect the relevance of features to classification. At each stage, alternatives were compared in terms of their performance in the context of a small sample. Differentiating sulcal patterns were located in temporal and precentral areas, as well as the collateral fissure. We also verified the benefits of applying dimensionality reduction techniques and validation methods, such as resubstitution with upper bound correction, to optimize performance.

## Graphical abstract

A CAD system using sucal features derived from brain structural is implemented though statistical, machine learning and deep learning techniques. Explainable AI techniques were applied to enhance the interpretability. Sulcal patterns in specific brain areas associated with schizophrenia, such as temporal and precentral areas, and the collateral fissure were identified.

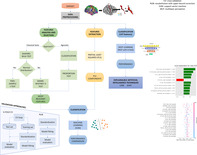

## 1 INTRODUCTION

This period of history is witnessing the breakthrough into clinical trials of computer‐aided diagnosis (CAD) systems based on artificial intelligence (Hope Weissler et al., 2021). Research of, and with CAD systems proliferate in the literature with applications in Alzheimer's disease (Graña et al., 2011; Javier Ramírez et al., 2013; Ortiz et al., 2016), Parkinson's disease (Arco et al., 2022; Martinez‐Murcia et al., 2018; Shu Lih et al., 2018) and autism (Leming et al., 2020; Leming et al., 2021; McAlonan, 2004). These approaches are primarily based on features extracted from imaging of brain function, with fMRI and PET, and brain structure, using MRI from which grey matter volumes can be estimated (Gorriz et al., 2021; Wright et al., 1999) or morphological features extracted from the cerebral cortex (Jimenez‐Mesa et al., 2020). A feature newly available from surface representations of the cortex are the sulcal (concave) and gyral (covex) folds (Campero et al., 2014).

Sulcal patterns offer particularly interesting features. They generally form in the last trimester and early life and remain broadly unaltered throughout adulthood, although the complex patterning of the cortex, whilst unique to the individual, strongly varies across individuals. They therefore potentially contain information about early development including the fetal and infant environment (Cachia et al., 2021). There are several approaches in the literature describing the detection, labelling and characterisation of sulci (Andreasen et al., 1994; Auzias et al., 2015; Beeston & Taylor, 2000; Behnke et al., 2003; Mateos et al., 2020; Yang & Kruggel, 2008). BrainVISA (Geffroy et al., 2011), is a software package which undertakes all these steps (Borne et al., 2020; Perrot et al., 2011). Other available packages include Freesurfer (https://surfer.nmr.mgh.harvard.edu) (Schaer et al., 2008) for detection and labelling combined with calcSulc (Madan, 2019) or BrainGyrusMapping (Murphy et al., 2014) for the calculation of characterising features of each sulcus. Each approach has its strengths and limitations (Mikhael et al., 2018).

Sulcal information has proven to be useful in the study of a wide range of conditions; for example, in Alzheimer's disease (Maciej Plocharski and Lasse Riis Østergaard, 2016; Mateos et al., 2020), Parkinson's disease (Wang et al., 2011), and anorexia (Collantoni et al., 2021; Wagner et al., 2003). Schizophrenia has a rich and well‐replicated literature establishing patterns of cortical change (Liu et al., 2020; Palaniyappan et al., 2018; Sallet et al., 2003; Zhang et al., 2012). Whilst there has been some work on both overall and specific sulcal information in schizophrenia (Csernansky et al., 2008; Janssen et al., 2022; Rollins et al., 2020), there has not, to our knowledge, been any exploration of the sulcal pattern as a way to classify individuals with schizophrenia from unaffected controls.

One of the main problems often encountered in conducting this type of study is the limited number of samples available. This is of particular concern when the number of features associated with each sample is very high; known as the *curse of dimensionality* (Gorriz et al., 2017). This is also a problem when applying classical statistics which make strong assumptions based on the sample conforming to the normal distribution. When the number of samples is small, it is not easy to accurately determine the distribution from which they are sampled and sometimes invalid techniques are implemented or inaccurate results are obtained (Eklund et al., 2016; Ioannidis, 2005). For this reason it is useful to consider other methods, such as data‐driven approaches (Gorriz et al., 2021) based on artificial intelligence, both machine learning and deep learning (Górriz et al., 2020). A key benefit is to obtain insights similar to those obtained by parametric statistical approaches but without requiring the dataset to satisfy certain conditions. Furthermore, the black box problem, whereby there is no easy interpretation of the biological meaning of a classification result or understanding of the underlying decision‐making process, is now being addressed with explainable artificial intelligence (XAI) algorithms (Gunning et al., 2019; Jimenez‐Mesa, Arco, et al., 2023; van der Velden et al., 2022).

In this study, we explore the capacity of measurements of sulcal patterns to discriminate between patients with schizophrenia and controls. Initially, features relevant to this classification problem were identified by traditional univariate statistical methods. The accuracy of classification was then compared between machine learning classifiers of varying complexity with input features from prior multivariate analysis of identified features. Explainable machine learning techniques were also deployed to give a richer description of the pattern of case–control differences observed.

This article is organised as follows. Section 2 provides a detailed description of the database and the preprocessing pipeline applied for extraction of sulcal features. Section 3 describes the methods applied in this study, including feature selection and extraction, classification algorithms, validation methods and XAI techniques, among others. Then, in Section 4 we evaluate the results obtained. Finally, outcomes and future study are discussed in Section 5, and conclusions are drawn in Section 6.

## 2 MATERIALS

### 2.1 Database

Data used in this study consisted of MRI from 65 (27 females) Han Chinese patients with schizophrenia (SCZ) and 57 (24 females) unaffected controls (HC). Participants were recruited from the Shanghai Mental Health Centre and the data set was published and analysed in (Li et al., 2018). All participants provided a written informed consent. The study was approved by the Ethics Committees of the Shanghai Mental Health Centre and the Institute of Psychology, the Chinese Academy of Sciences.

Participants underwent structural neuroimaging as well as a clinical evaluation. High‐resolution T1‐weighted structural images were acquired on a 3T MRI scanner with 1 mm isotropic voxel size. Details of the acquisition parameters are given elsewhere (Li et al., 2018). No non‐linear spatial normalization was applied to the scans to avoid possible bias generated from shape deformations of the sulcal patterns (Cachia et al., 2008; Mellerio et al., 2016).

### 2.2 Data preprocessing

**FIGURE 1:**
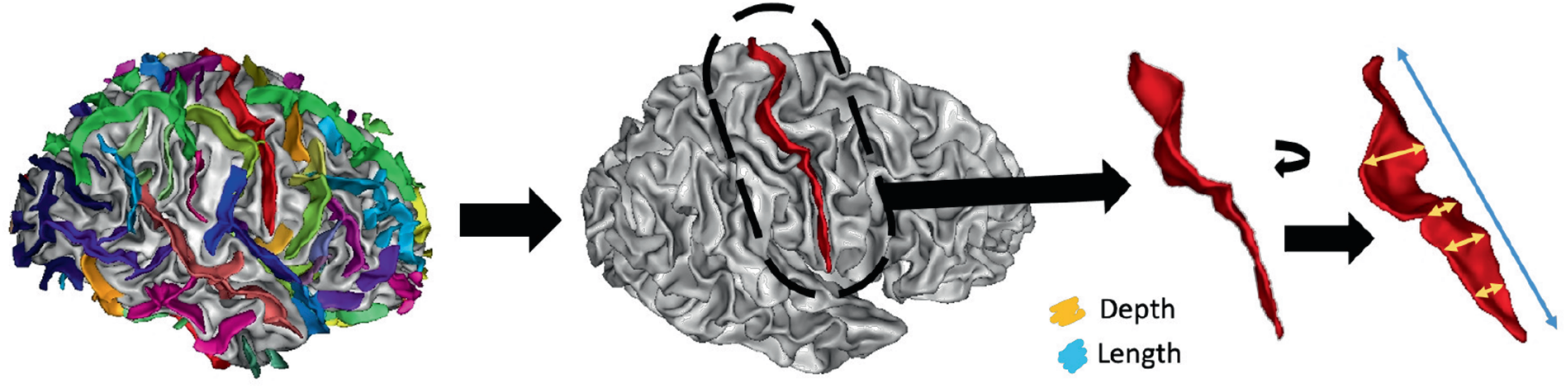
Example of a brain with sulci regions automatically labelled by BrainVISA using Morphologist 2021 pipeline (right). The central sulcus is highlighted (middle) and indicates how length and depth are measured in a region (left).

The MRI scans were processed using BrainVISA 5.0.4 (Geffroy et al., 2011) to extract sulcal features by means of the Morphologist 2021 pipeline (Borne et al., 2020; Perrot et al., 2011). Information was obtained from 62 areas per hemisphere (123 in total; the sulcus of the supra‐marginal gyrus is only defined in the left hemisphere). In each region, the features measured in Talairach space (Louis Collins et al., 1994; Talairach, 1988) were length, depth (average and maximum), fold opening, medial surface of the cortical folds and grey matter thickness (Jin et al., 2018; Pizzagalli et al., 2017). The average and maximum depth and length were calculated as features. Other available features are associated with morphological parameters rather than surface topology. Figure 1 shows an example of an automatically labelled brain by BrainVISA and the features extracted from a specific region.

**FIGURE 2:**
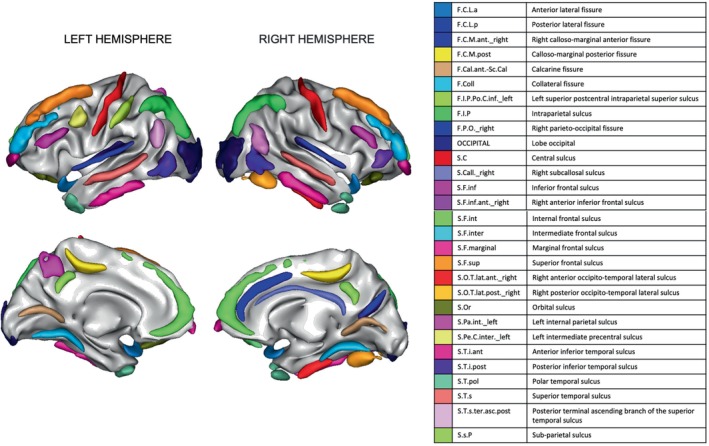
The 49 regions from the BrainVISA sulcal atlas (Perrot et al., 2011) used in this study. All other regions were excluded due to sulcal misdetection.

In some cases, a particular sulcus could not be identified or was misdetected by the Morphologist 2021 pipeline. Therefore, samples with more than 18 of these events (15% of the total number of regions) was excluded from the analysis. Insula (left and right) regions were also excluded because of the high possibility of being misdetected due to its peculiar shape. After these exclusions, any region that still had at least one misdetection across remaining participants was excluded. Finally, features were normalised to zero mean and standard deviation 1. Individuals with any feature with values >6 times the standard deviation were removed. These exclusion criteria result in 49 remaining areas for analysis, which are shown in Figure 2. The final number of individuals (samples) was 114, the demographics for whom are shown in Table 1. It can be seen that the sample set was matched for size, sex and age, with a sample size of 58 SCZ patients and 56 HC.

**TABLE 1:** Demographic details of the participants included in the analysis.

	SCZ	HC	Total	*t*/$\chi^{2}$ statistic	*p* value
Number	58	56	114		
Sex (Male/Female)	35/23	29/27	64/50	0.85	.357
Age	22.95 ± 5.64	24.79 ± 7.36	23.85 ± 6.57	1.20	.233
IQ	93.18 ± 18.40 (N = 55)	116.41 ± 14.38	105.30 ± 19.91	7.16	<.0001*
Years of education	12.41 ± 2.91	13.40 ± 2.54	12.90 ± 2.77	1.93	.056
Hallucinations (yes/no)	20/38	0/56	20/94	76	<.0001*

*Note*: *p* values were obtained using: two‐sample *t*‐test or Chi‐Square test, * when p<.05.

Abbreviations: HC, healthy controls; IQ, Intelligence quotient; SCZ, patients with Schizophrenia.

## 3 METHODS

### 3.1 Feature analysis and selection

Following preprocessing described in Section 2.2, sulcal length and maximum and mean sulcal depth were tested by univariate statistical methods to identify features important to classification. Both parametric and non‐parametric techniques were considered.

#### 3.1.1 Parametric techniques

Initially, the Shapiro–Wilk test (Shapiro & Wilk, 1965) was applied to identify which features obeyed a normal distribution, since the null hypothesis is that samples come from a normally distributed population.

For those features where a normal distribution was followed, a two‐sample t‐test (Kim, 2015; Welch, 1947) was applied to detect the relevance of the feature to distinguish between schizophrenia and control participants. To compare the importance of features, the *p* values (Panagiotakos, 2008) associated with the tests were used.

Those features that did not follow a normal distribution were assessed with the Mann–Whitney *U* test (Fay & Proschan, 2010; Mann & Whitney, 1947), and the corresponding *p* value used.

#### 3.1.2 Non‐parametric techniques

The importance of a feature to classification was also evaluated by means of an AI‐based approach: Statistical Agnostic Mapping (SAM) (Gorriz et al., 2021). First, each feature was independently fed into a supervised classification model. Then, accuracies obtained for each feature were sorted based on a proportion test. The null hypothesis of the test is that the population proportion is similar to a particular proportion, $\pi_{0}$, given a confidence interval. The test statistic for each feature was estimated as:

(1) $$z=\frac{\hat{\pi }-\pi_{0}}{\sigma_{0}}$$

where $\hat{\pi }$ is the accuracy related to the feature, and $\sigma_{0}=\sqrt{\left(\pi_{0}\left(1-\pi_{0}\right)\right)/l}$. In this last expression, l is the number of accuracies higher than $\pi_{0}$. In our case, $\pi_{0}$ is the mean of all features' accuracies, both empirical (derived from samples) and actual (related to an infinite sample set). Once the z‐statistics were calculated, the *p* value of each statistic was estimated. For this, the null hypothesis was considered to be true and therefore the test statistic follows a standard normal distribution. From this *p* value, the most relevant features of the study were determined.

### 3.2 Feature extraction

Along with feature selection, features were also processed to generate more compact information and reduce the dimension of the feature vector. Partial Least Squares (PLS) (Wold et al., 1984) is a supervised method which allows dimensionality reduction while retaining the patterns for higher separability of the classes. Given a matrix of features, $X_{lxm}$, where l is the number of samples and m the number of features, and a vector of labels$Y_{lx1}$, PLS generates a matrix of loadings $X_{l}$, which is related to the initial data by the following linear combination:

(2) $$X=X_{s}X_{l}^{T}+E$$

where $X_{s}$ is the score matrix and E the assumed error matrix. The reduced d‐dimensional space desired comes from the dimensions of $X_{l}$ (m x d), as m>d. This new reduced space contains the original information of X.

### 3.3 Classification

Once the features to undertake the classification were selected, the next stage was classification. For the binary classification problem posed in this study, both machine learning (ML) and deep learning (DL) methods were applied.

The ML algorithm implemented in this study was a Support Vector Machines (SVM) classifier with linear kernel (Schölkopf & Smola, 2002). This combination was chosen for its easy explainability as well as its propensity to generate excellent results in neuroimaging (Javier Ramírez et al., 2013; Jimenez‐Mesa et al., 2020; Orru et al., 2012). This supervised algorithm establishes the maximum‐margin hyperplane which separates the samples of the different classes. In the case of a linear binary problem, the set of points, x, that generate the hyperplane satisfy:

(3) $$w^{T}x-b=0$$

where w is the normal vector to the hyperplane and b represents the error. The classification is done in such a way that samples on one side of the hyperplane belong to one class, and samples on the other side are associated with the second class.

The selected DL architecture was a multilayer perceptron (MLP) as both the number of samples and features was small. Additionally, a two‐dimensional vector favours MLP over convolutional neural network (CNN). The MLP is a feedforward artificial neural network (ANN) composed of fully connected layers. Each layer has i perceptrons which are connected in a forward direction to the perceptrons of the next layer, but with no connections between perceptrons of the same layer. Given a layer n, the output value of each perceptron is computed as:

(4) $$y_{i}^{n}=f\left(w_{i}^{n}\cdot y^{n-1}+b_{i}^{n}\right)$$

where $f\left(\cdot \right)$ is the activation function applied to the i‐th perceptron. This function is applied to the result of multiplying the weight vector, $w_{i}^{n}$, and the activations of the previous layer, $y^{n-1}$, in addition to an associated bias, $b_{i}^{n}$.

**FIGURE 3:**
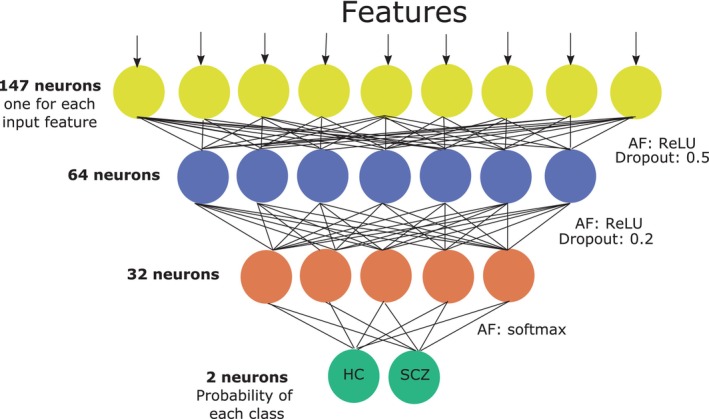
Scheme of the MLP composed of four layers: input layer, two hidden layers and the output layer. AF, activation function.

The network configuration implemented is shown in Figure 3. The number of epochs involved in the training was 18, with a batch size of 1. The optimizer selected was Adam with a learning rate of 0.001, and the stopping criterion computed as the cross entropy loss with balanced weights.

### 3.4 Validation procedure

Two validation methods were used to assess the performance of the classifiers. First, a 10‐fold stratified cross‐validation scheme (Kohavi, 1995) was applied, which guaranteed independence between training and test samples. The sample was randomly divided in to a set in 10 folds, and for 10 iterations used one of the folds as test samples and the remaining folds as the training set. For the computation of the performance metrics, the mean and standard deviation of the values obtained in the 10 iterations were used.

The second validation method was an upper bound‐corrected resubstitution (Vapnik et al., 1994), which is referred to in previous work as RUB (Jimenez‐Mesa, Arco, et al., 2023; Jimenez‐Mesa, Ramirez, et al., 2023). One way to define the upper bound would be as the difference between empirical and actual errors given a fitted learning algorithm, $\mu \geq |E_{\mathrm{act}}\left(f\left(x\right)\right)-E_{\mathrm{emp}}\left(f\left(x\right)\right)|$, or in terms of the previous approach, the difference between the training error and the test error. Thus, the entire database was used as the training set for the classifier, that is, resubstitution was performed, and then the actual accuracy was obtained by means of the upper bound. This could be considered a theoretical classification limit that allows the use of all accessible data to compute the metrics of interest. In addition to accuracy, other metrics such as sensitivity or specificity can also be of limited value since their errors are related to the classification error.

Different upper bounds are described in the literature. The most well‐known is based on the VC dimension as proposed by V. Vapnik (Vapnik et al., 1994). In this article, an upper bound based on the assessment of concentration inequalities was applied (Górriz et al., 2019). This bound is only applicable to linear classifiers, for example, SVM with linear kernel, and its expression is:

(5) $$\mu_{\mathrm{emp}}\leq \sqrt{\frac{1}{2n}\ln\frac{2\sum_{k=0}^{d-1}\left(\begin{array}{c} n-1\\ k \end{array}\right)}{\eta }}$$

where n is the number of samples used, size of the sample set, d is the feature's dimension, and η is the significance level. In this study, the significance level was set as 0.05.

The implementation of probably approximately correct (PAC)‐Bayesian bounds is another interesting proposal. In this study, a dropout bound (McAllester, 2013) was analysed. This bound considers a dropout rate, $\alpha \in \left[0,1\right]$, which reduces the complexity cost of the function. The effect of this dropout is stronger the closer its value is to 1. The expression of this bound in the scenario proposed in this study is:

(6) $$\begin{array}{l} \mu_{\mathrm{PAC}-\text{bayes}}=\min_{1\leq i\leq k}\;\left(\frac{1}{1-\frac{1}{2\lambda_{i}}}-1\right)\hat{L}\left(Q\right)+\\ \;\frac{1}{1-\frac{1}{2\lambda_{i}}}\left(\frac{\lambda_{i}L_{\max}}{n}\left(\frac{1-\alpha }{2}∥\Theta {∥}^{2}+\ln\frac{k}{\eta }\right)\right) \end{array}$$

where k different values of the parameter λ, which was set to 1/2≤λ≤10, were evaluated to minimise the bound. The estimated value of the loss function to be bounded, that is, the error of the classifier, is $\hat{L}\left(Q\right)$. Its maximum value, $L_{\max}$, which must be a real number, is 1 in this case. Finally, Θ was the classifier's parameter set.

### 3.5 Explainable AI

Algorithms that give a qualitative understanding of performance are key to extracting domain information from classification tasks. This emerging field is referred to as explainable artificial intelligence (XAI). Here, apart from considering the performance of the classifier, two of these techniques were used to analyse the influence of features on the decision making by the classifiers.

Local Interpretable Model‐agnostic Explanations (LIME) (Ribeiro et al., 2016) focuses on providing explanations of individual predictions of the classifier model. To do so, it makes a local approximation to an easily interpretable model. Given the type of data used in this study, LIME highlights the most relevant, both positively and negatively, sulcal features during classification. In other words, LIME shows if a high value of a feature brings the sample closer to a class (acts positively) or reduces the likelihood of the sample belonging to that class (acts negatively).

This algorithm is able to explain any prediction model f locally. This means that LIME provides explanations for a particular sample x, since globally faithful explanations are still a challenge for complex models (Ribeiro et al., 2016). To do this, the algorithm selects an explanation model g∈G, where G is a class of potentially interpretable models. The selection is made according the following objective function related to the faithfulness of the explanation model:

(7) $$\xi =\text{argmin}\;ℒ\left(f,g,\pi_{x}\right)+\Omega \left(g\right)$$

where interpretability and local fidelity is ensured by minimising the trade‐off between the loss related to the discrepancy between g and f given the local kernel $\pi_{x}$, and the complexity of g, measured by $\Omega \left(g\right)$.

SHapley Additive exPlanations (SHAP) (Lundberg & Lee, 2017) is a model‐agnostic algorithm which can explain any classification model. SHAP assigns the relevance of each feature by means of Shapley values, a concept from game theory (Shapley, 1953).

Given the set of features S, the contribution of each feature s is estimated on the basis of its average marginal contribution to all subsets of features T⊆S, which do or do not include the feature s. Let the prediction of the model given a particular sample and a subset of features be denoted as $f_{x}\left(T\right)$. The marginal contribution of the feature s is estimated as the difference in predictions when applying or not applying such a feature, $\left[f_{x}\left(T\cup s\right)-f_{x}\left(T\right)\right]$. So, the Shapley value, $\phi_{s}$, is computed considering all possible subsets $T\subseteq S\backslash \left\{s\right\}$:

(8) $$\phi_{s}\left(f,x\right)=\sum_{T\subseteq S\backslash \left\{s\right\}}\frac{\left|T\right|!\left(\left|S\right|-\left|T\right|-1\right)!}{\left|S\right|!}\left[f_{x}\left(T\cup s\right)-f_{x}\left(T\right)\right]$$

SHAP values are the solution to Equation (8), that is, they are Shapley values of a conditional expectation function of the original model which satisfy properties such as local accuracy, missingness and consistency (Lundberg & Lee, 2017). Several approximation methods to compute SHAP values are proposed, since its exact computation is difficult to achieve. The one applied is this study was Kernel SHAP, a model‐agnostic approximation which combines Shapley values and linear LIME (local linear regression) to estimate the importance of each feature. To do this, the solution of Equation (7) are Shapley values; that is, local accuracy, missingness and consistency must be satisfied. Inherently, LIME does not meet all these properties by choosing its parameters heuristically.

Both techniques have generated interesting results in previous neuroimaging studies (Lombardi et al., 2021; Lombardi et al., 2022; Scheda & Diciotti, 2022), where their application allows observation of the congruence of the explanations and their usefulness.

### 3.6 Performance evaluation

Performance of the classifiers was evaluated through metrics extracted from the confusion matrix, where the positive class was SCZ. These metrics were balanced for accuracy, specificity and sensitivity. Their equations are:
(9)

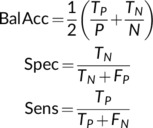

where $T_{P}$ refers to the number of participants correctly classified as SCZ (true positives), $T_{N}$ corresponds to the number of controls correctly identified (true negatives), $F_{P}$ quantifies the number of controls misclassified (false positives), and $F_{N}$ quantifies the number of SCZ participants misclassified (false negatives).

The receiver operating characteristic (ROC) curve was also constructed. The area under the ROC curve (AUC) evaluates the ability of the model to differentiate between the two classes (Hajian‐Tilaki, 2013; Mandrekar, 2010).

### 3.7 Summary of the procedure

**FIGURE 4:**
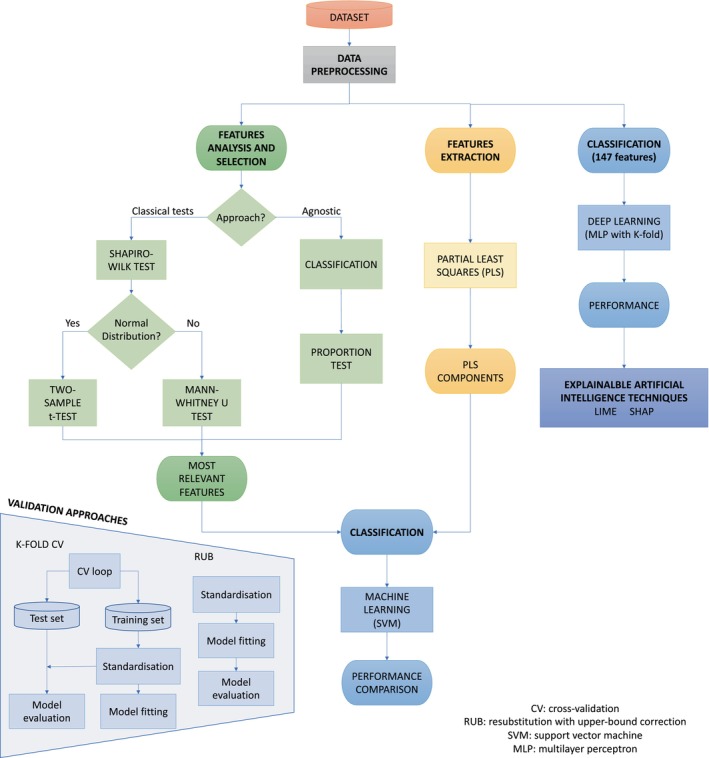
Flowchart of the study. After preprocessing the data, two independent feature selection and feature extraction analyses were conducted. The information extracted from both was fed into a ML classifier. Two validation methods were applied. Finally, the 147 preprocessed features are fed into a multilayer perceptron (ML) model. The classifiers' performance was analysed by means of XAI techniques.

The several stages of this work are depicted in Figure 4. In summary, once data were preprocessed as described in Section 2.2, two different scenarios were implemented: feature selection (see Section 3.1), which highlighted the relevance of sulcal features, and feature extraction (see Section 3.2), which generated a reduced set of features to make the best possible use of the information extracted from the original data. From the features highlighted or generated by both approaches, a classification stage followed where a ML model (SVM) was applied using various validation methods, as described in Sections 3.3 and 3.4. Lastly, the classification model (MLP) obtained when the 147 preprocessed features were used was analysed by means of the XAI described in Section 3.5.

## 4 RESULTS

Three respective features (length, mean depth and maximum depth) were extracted from 49 brain regions. The total number of features in this study was 147, a value larger than the number of samples, 114. This is an undesirable, but very common situation in neuroimaging.

### 4.1 Parametric feature selection

**FIGURE 5:**
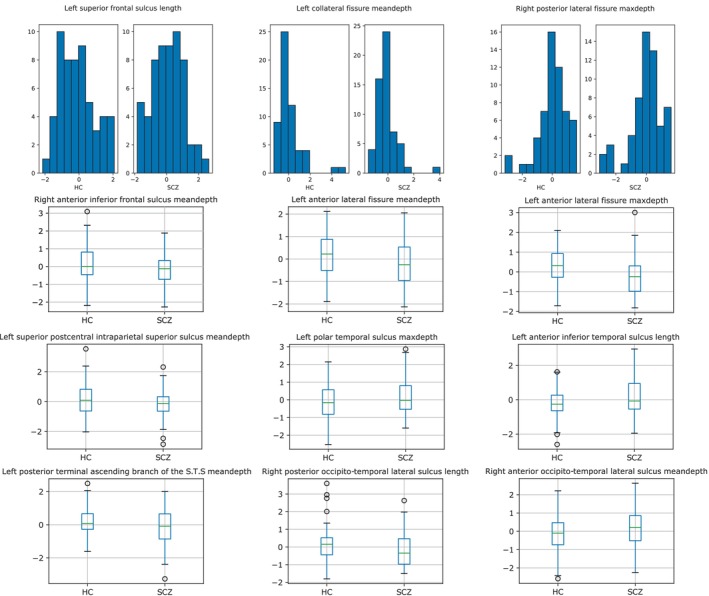
Statistical features analysis. Top row: histograms related to non‐normal distributed features. Bottom row: boxplots of the nine most relevant features according to the Two‐sample t‐test and the Mann–Whitney *U* test, depending on whether the feature follows a normal distribution or not. These features are arranged from Frontal lobe to Occipital lobe (from left to right and from top to bottom). Average and maximum depth are abbreviated as meandepth and maxdepth.

To analyse the relevance of the features, firstly we followed a parametric approach. The Shapiro–Wilk test for normality determined that among the 147 features 125 followed a normal distribution, while the remaining 22 did not. The top row of Figure 5 shows examples of histograms of three features that did not follow a normal distribution. It can be seen that the main reason for this was the long tails skewing the distribution. By visual inspection selecting eligible samples, the number identified was adequate for a two‐sample t‐test with all the features. The Mann–Whitney U test was used for non‐normally distributed features.

The significance of each feature was assessed with the *p* value obtained in their respective tests. The bottom graph in Figure 5 shows a boxplot of the nine most relevant features according to the tests applied. Only the first five had a *p* value <0.05, while 10 of them had a *p* value <0.1.

### 4.2 Non‐parametric feature selection

**FIGURE 6:**
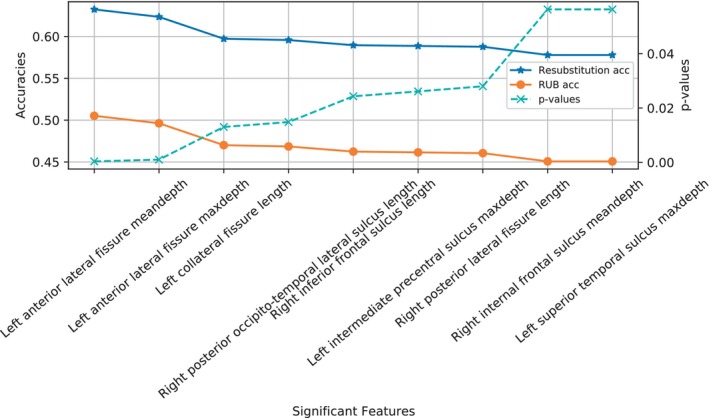
The nine most significant features obtained by a classification approach. Their related accuracy was estimated as the mean value of 1000 permutations shuffling the samples and using a SVM with lineal kernel classifier and resubstitution with upper bound correction (RUB) as a validation approach. Resubstitution accuracy stands for the empirical accuracy obtained before the upper bound is applied. The *p* values related to each region were estimated using a test of a proportion. Average and maximum depth are abbreviated as meandepth and maxdepth.

Using the SVM algorithm with linear kernel and a validation based on the resubstitution with upper bound correction, 1000 permutations were applied by randomly modifying the position of the samples in the set and calculating the mean accuracy for each feature. For accuracy estimation, a balanced estimator with class weights balancing was applied during the classifier training. The *p* value associated with each feature was assessed with a significance test for a proportion. The nine most relevant features obtained are shown in Figure 6.

### 4.3 Most relevant features analysis

**FIGURE 7:**
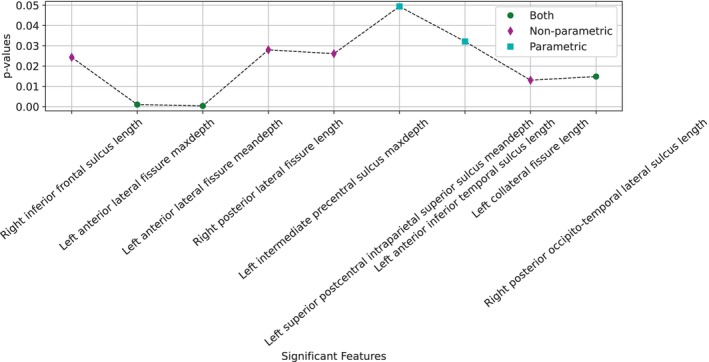
Features under analysis with a *p* value <0.05 in any of the parametric and non‐parametric tests. These features are arranged from Frontal lobe to Occipital lobe. The significant features under the parametric analysis are coloured cian, non‐parametric analysis are coloured magenta, or if both they are coloured green. Average and maximum depth are abbreviated as meandepth and maxdepth.

Figure 7 shows the most relevant features ranked by their *p* value in both approaches (parametric and non‐parametric). Relevant features had a *p* value <0.05, identifying nine features. Three features appeared with both approaches, together with two from the parametric and four from the non‐parametric approach. These include both depth‐related and length‐related features.

### 4.4 Use of reduced dimensionality in classification

**FIGURE 8:**
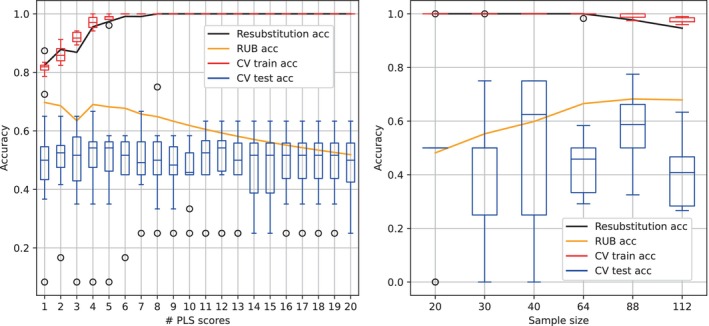
Left: Performance of the SVM classifier along with PLS as the feature extraction technique. Results are shown for a wide range of PLS components (1–20). Right: performance of the SVM classifier using four PLS components for several balanced samples sizes (20, 30, 40, 64, 88 and 112). In both cases: RUB (orange line) and 10‐fold CV (box‐plots). Resubstitution accuracy (black line) stands for the empirical accuracy obtained before the upper bound is applied.

Instead of analysing the relevance of the features independently, it is possible to analyse the relevance of the feature set for the case–control classification. To do this, a feature extraction stage was implemented by applying PLS to the original data. The reduced feature dimension was then classified with a SVM classifier with a linear kernel. Performance was analysed using cross‐validation (K‐fold) and RUB as validation procedures. Figure 8 (left) shows results how the classifier's performance varied according to the PLS components used. The upper bound applied in RUB, as detailed in Equation (5), depends on the number of features, the fixed number of samples (114) and significance level (0.05). Although there were no discernible trends in performance as a function of number of PLS scores using K‐fold, a decreasing trend was observed applying RUB and higher accuracies were obtained using fewer components (<6). Conflating both approaches, four PLS components were selected and results are illustrated in Figure 8 (right).

### 4.5 Impact of sample size

To understand the effect on performance of sample size, four PLS components were chosen as input features for the classifier. The results are shown in Figure 8 (right). Theoretically, accuracy should increase as the sample is enlarged, but this was not the case with K‐fold. The upper bound applied in RUB changes according to the number of samples, with a fixed number of features, 4, and a significance level, 0.05.

### 4.6 Various classification scenarios

Case–control classification was undertaken with the features selected with the parametric, non‐parametric and ensemble approaches as well as PLS features. The testing of the classification results were obtained by performing 1000 permutations of the dataset, which are shown in Table 2. Note that for the computation of the upper bound of Equation (5), the RUB validation approach took into account the number of samples, 112 (as the data was balanced in each iteration), the number of features (9 or 4, depending on the case), and the significance level (0.05). This gave values for the upper bound of 0.3695 per unit or 36.95% for nine features and 0.2675 (26.75%) for 4. The reader is reminded that these values must be subtracted from the accuracy rate obtained in order to determine the actual worst‐case accuracy rate. While K‐fold CV worked reasonably well with the extracted features, especially with those obtained with the parametric method, RUB had improved performance with the PLS components due to fewer input features, and thus a tighter upper bound. This is especially true when extracting the main components of the full set of features.

**TABLE 2:** Performance of the SVM classifier using the nine extracted features in the parametric, non‐parametric and both analyses after 1000 permutations.

		Parametric	Non‐parametric	Both	PLS (all)	PLS (both)
10‐Fold training	Acc (%)	73.59 ± 0.96	71.25 ± 0.83	71.19 ± 0.78	97.18 ± 0.56	70.82 ± 0.82
	Sens (%)	67.98 ± 1.31	66.21 ± 1.42	67.72 ± 1.38	97.37 ± 0.67	67.80 ± 1.32
	Spec (%)	79.20 ± 1.08	76.28 ± 1.50	74.68 ± 0.98	96.98 ± 0.75	73.85 ± 1.10
	AUC	0.80 ± 0.01	0.76 ± 0.01	0.76 ± 0.01	1.00 ± 0.00	0.76 ± 0.01
10‐Fold test	Acc (%)	66.26 ± 2.37	62.32 ± 2.72	64.45 ± 2.30	49.64 ± 3.04	63.12 ± 2.48
	Sens (%)	62.07 ± 2.80	59.65 ± 3.50	61.94 ± 3.03	50.08 ± 4.39	62.00 ± 3.07
	Spec (%)	70.44 ± 3.48	64.97 ± 4.02	66.92 ± 3.30	49.23 ± 4.05	64.25 ± 3.66
	AUC	0.73 ± 0.02	0.67 ± 0.03	0.68 ± 0.03	0.48 ± 0.03	0.66 ± 0.03
RUB	Acc (%)	34.38 ± 1.44	34.96 ± 1.41	33.34 ± 1.22	68.88 ± 1.15	43.77 ± 1.60
	Sens (%)	27.51 ± 1.79	29.82 ± 1.86	30.88 ± 2.37	69.77 ± 1.48	40.64 ± 2.30
	Spec (%)	41.25 ± 1.52	40.11 ± 2.86	35.81 ± 2.48	67.99 ± 1.54	46.90 ± 2.43
	AUC	0.43 ± 0.01	0.39 ± 0.01	0.38 ± 0.01	0.73 ± 0.00	0.49 ± 0.01

*Note*: Results using four PLS components as input to the classifier are also included when they are extracted from all 147 and the nine globally significant ones. Upper bounds related to this analyses were 0.3695 (9 features) and 0.2675 (4 features) for a significance level of 0.05.

### 4.7 Comparison of upper bounds

**FIGURE 9:**
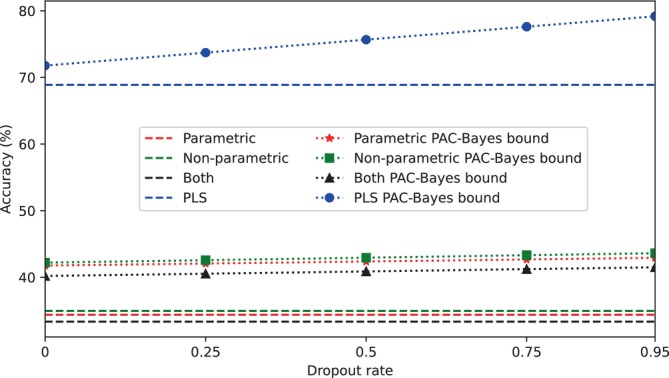
Accuracies obtained with the RUB approach using two different upper bounds. The dashed horizontal lines are the accuracies obtained with the upper bound based on concentration inequalities (Equation (5)). Accuracies with markers are those with the PAC‐Bayes bound (Equation (6). The classifier applied was SVM using the nine extracted features in the parametric, non‐parametric and both analyses, and four PLS components. Accuracies shown are the mean values after 1000 permutations.

A PAC‐Bayes upper bound was applied under the same experimental conditions to test its performance against the upper bound based on concentration inequalities. As this different bound depends on the dropout rate, see Equation (6), several values of dropout were applied: 0, 0.25, 0.5, 0.75 and 0.95. The results are shown in Figure 9, where the dashed horizontal lines represent the value shown in Table 2 with the RUB approach.

### 4.8 Examining predictions with XAI

The same classifier was tested using the 147 features as input features. A summary of the performance results are shown in Table 3. Accuracy values were below 50%. With the same features as input, the MLP achieved a 58.83% accuracy on the test set by applying CV. By applying all the features as input, we observed from explainable artificial intelligence techniques the main focus of the algorithm. Due to the better performance obtained using MLP, the subsequent results are associated with this classification model.

**TABLE 3:** Classification performance of models based on SVM and MLP when the 147 features (the complete set) were fed as input of the classifier. Cross‐validation was used as validation approach (10‐Fold CV).

	SVM	MLP
10‐Fold training		
Acc (%)	100 ± 0.00	67.94 ± 8.90
Sens (%)	100 ± 0.00	67.04 ± 26.06
Spec (%)	100 ± 0.00	68.83 ± 21.01
AUC	0.40 ± 0.49	0.71 ± 0.07
10‐Fold test		
Acc (%)	49.50 ± 9.72	58.83 ± 6.28
Sens (%)	54.00 ± 17.50	57.00 ± 27.87
Spec (%)	45.00 ± 14.47	60.67 ± 28.43
AUC	0.45 ± 0.13	0.56 ± 0.10

#### 4.8.1 LIME

**FIGURE 10:**
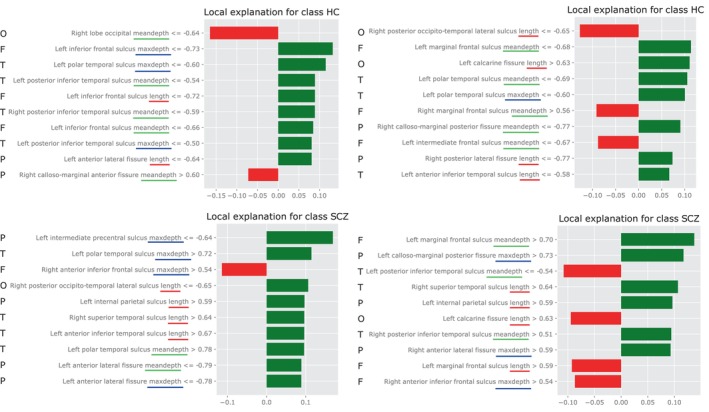
Local explanations extracted from LIME for the schizophrenia (SCZ) and control (HC) classes. Features in green represent values that increase the chance of being classified as the class under analysis. Features in red reduce it. Top row: explanations for two correctly classified HC test samples. Bottom row: explanations for two correctly classified SCZ test samples. To improve comprehensibility, length, mean depth and maximum depth are underlined in red, green and blue, respectively. On the left side, the letters F, T, P and O represent the feature belonging to Frontal, Temporal, Parietal or Occipital lobe, respectively. Average and maximum depth are abbreviated as meandepth and maxdepth.

LIME allowed us to identify qualitative patterns on the most relevant features according to a classifier that distinguished case and control classes. Four examples of individual explanations are shown in Figure 10. These examples are related to correctly classified HC (top row) and SCZ (bottom row) test samples by the MLP. For this analysis, the 10 most relevant features in the classification for each sample were selected and displayed sorted from most to least importance according to LIME.

All four major lobes of the brain appear in this analysis, although the Temporal and Frontal lobes have greater representation. The same applies to the three types of features related to length and depth. Several features included in Figure 7 as the most relevant features, according to parametric and non‐parametric approaches, were also relevant in this analysis. One prominent example was the length of right posterior occito‐temporal lateral sulcus. In the top right sample, a low value of the length of right posterior occito‐temporal lateral sulcus decreased the probability of being associated to HC class, while in the bottom right sample, a similar low value increased the chance of being classified as a SCZ patient.

#### 4.8.2 SHAP

**FIGURE 11:**
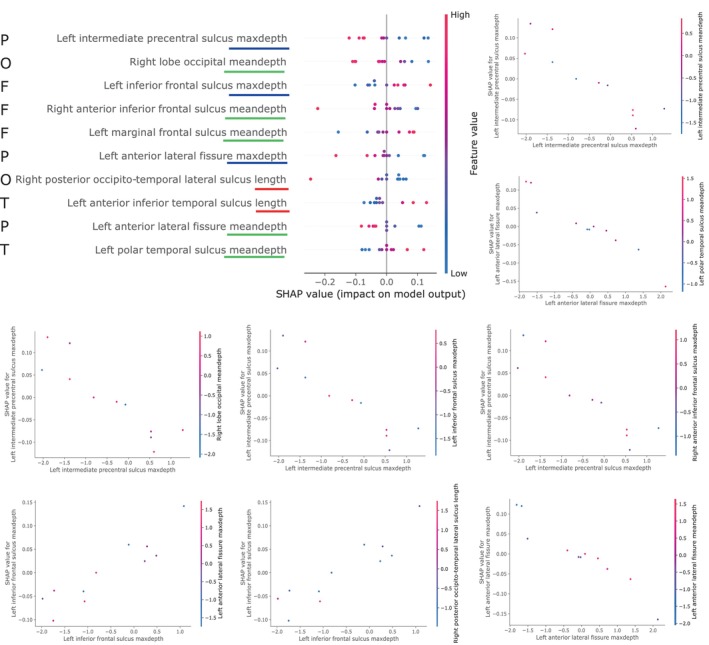
SHAP charts, where each point represents an instance of the test sample. Top left: Summary plot of features importance in the classification decision; the 10 most relevant are shown. To improve comprehensibility, length, mean depth and maximum depth are underlined in red, green and blue, respectively. Letters F, T, P and O represent the feature belonging to Frontal, Temporal, Parietal or Occipital lobe, respectively. Top right and bottom: Dependence plots of some relevant regions according to their SHAP values. Colour in the graph corresponds to the value of a second feature for that same sample. The positive class is SCZ. Average and maximum depth are abbreviated as meandepth and maxdepth.

Figure 11 shows the graphs that this technique returned from the MLP model. Figure 11 (top left) is a summary graph of the impact of the features during the classification process. The 10 most relevant features in the classification are displayed. High SHAP values were associated with the SCZ, while low values were associated with HC. Colour blue in the instances of the test sample indicates low values of the feature whereas a pink value indicates the opposite. Please refer to the supplementary material for a summary graph with the 147 features.

Overall, mean and max depth of specific sulci are notable in their contribution to the classification. In this analysis, the impact of length is minor. As examples, a high value of the maximum depth of left intermediate precentral sulcus was associated with the HC class, since it was associated with lower SHAP values, whilst a high value of marginal frontal sulcus mean depth was associated with the SCZ class.

Figure 11 also includes several dependency plots of these most relevant features. Dependency plots illustrate the relationship between the SHAP value and the magnitude of the feature. A second feature reflected in the colour of the samples is included, which may indicate some dependency between features. At the bottom, it is observed that higher values of left inferior frontal sulcus maximum depth, that is, deeper values, bring the sample closer to the SCZ class (higher SHAP value). In the last graph, which includes the comparison between left anterior lateral fissure maximum and mean depth, there is a correlation between the two features, since samples with low values of the maximum depth also have a lower mean depth.

## 5 DISCUSSION

In this study, a staged approach of statistical, ML, and DL techniques were applied to perform an analysis of sulcal patterns in a case–control comparison of schizophrenia. Feature calculations were performed by BrainVISA, where a 3D U‐Net convolutional neural network was implemented to the labeling of sulci (Borne et al., 2020). Subsequently, sulcal length and depth were selected as features. These features were standardised and independently tested with parametric (t‐test) and non‐parametric (data‐driven) approaches. Machine and deep learning algorithms were applied to classify SCZ patients from HC, and its predictions are evaluated by XAI techniques.

Unlike most work on sulcus patterns, the features applied in this study are extracted fully automatically and encompass the entire cerebral cortex. The sulcal dectection processes remain in their early development, as it is still very difficult to correctly label all the sulcal patterns, especially those that are small or peculiarly shaped (Maciej Plocharski and Lasse Riis Østergaard, 2016). In the dataset used, the amount of detection failures obtained was high, thus reducing the number of sulci and the number of subjects finally included in the study. This made it impossible to study some high‐interest regions such as the left hemisphere paracingulate sulcus (Rollins et al., 2020), while the right hemisphere pair is represented in Figure 1 as right calloso‐marginal anterior fissure. Moreover, it was impossible to include data from other centres because even with standardised MRIs, similar values for the extracted features were not achieved. For these reasons, the literature includes a large number of works which combine automatic extraction and manual revision (Janssen et al., 2022; Liu et al., 2020; Shen et al., 2018), apply manual segmentation (John et al., 2006) or reduce the study to a concrete number of regions of interest (Jin et al., 2018; Yang et al., 2019).

Most significant features obtained in this study reflect a similar importance of length and depth, as it can be seen in Figures 5, 6, 7, albeit slightly higher in the case of depth. Regarding the relevance of using the maximum or mean depth, their occurrence in the most significant features is practically identical. However, given the same feature, both are not necessarily equally relevant. According to the upper right graph in Figure 11, while the correlation between maximum depth of the intermediate precentral sulcus and its effect on classification is inversely proportional, the mean depth has no direct relationship with maximum depth.

The hemisphere most represented in these findings is the left hemisphere, which is consistent with other studies (Cachia et al., 2008; Liu et al., 2020; Ribolsi et al., 2014; Rollins et al., 2020). Both the length and depth of the sulci in this hemisphere tended to be smaller in SCZ subjects, as observed previously (Cachia et al., 2008). For example, in line with previous studies, Figure 11 (left) shows a negative correlation between the intermediate precentral sulcus and the disease (Nesvåg et al., 2014; Palaniyappan et al., 2014). Nevertheless, differences were also found in the right hemisphere, which is aligned with hemispheric symmetry previously discussed in the literature (Csernansky et al., 2008), and as can be seen in Figure 10, where both left and right values were relevant in the classification. Nevertheless, there is something noteworthy in the relevance of the temporal region and that is that there was no decrease in the length values of this region for those from the SCZ class. There was a decrease in the value of maximum depth in the superior temporal sulcus in SCZ patients, which is consistent with previous study (Rollins et al., 2020). In fact, this feature is one of the most relevant obtained in the non‐parametric approach, see Figure 6.

Several other features associated with the temporal cortex can be seen in Figure 5. Of these, only the posterior terminal ascending branch of the superior temporal sulcus (S.T.s.ter.asc.post) had a lower average value for SCZ samples. This is also seen in Figure 11 by the association of high values of inferior temporal sulcus features with the SCZ class. On the contrary, the length of posterior occito‐temporal lateral sulcus was associated with smaller values for the SCZ class, see Figure 10.

As mentioned above, one of the most important regions for the study of schizophrenia is the medical surface of the brain around the cingulate sulcus (Garrison, Fernyhough, McCarthy‐Jones, Haggard, Carr, et al., 2015; Yang et al., 2019; Yücel et al., 2002). However, it is impossible to draw clear conclusions about this area in this study due to the elimination of most of its features at the preprocessing stage by the failure of the sulcal detection software. It is possible that this occurred because the surface morphology of this region varies greatly from one subject to another making it difficult to classify automatically. For this reason, the literature tends to undertake manual detection of this sulcus (Garrison, Fernyhough, McCarthy‐Jones, & Haggard, 2015; Rollins et al., 2020).

The limitations of this study include the reduced number of samples available. With a larger sample size, the results obtained could be strengthened and subtle changes in sulcal dimensions could be analysed in more detail. This is especially important when applying deep learning, as shown in its performance in Table 3. When introducing the 147 features, the network, although not excessively complex, was not able to obtain robust classifications due to a lack of samples. Therefore, in order to optimise the information extracted from the available data and to avoid the *curse of dimensionality* (samples vs. features ratio) (Gorriz et al., 2017), in addition to the widely used cross‐validation, resubstitution with upper‐bound correction was also adopted (Jimenez‐Mesa, Ramirez, et al., 2023; Vapnik, 1982).

This approach allows better performance to be obtained in small sample sizes, especially when the number of features is very small (ideally 1) (Castillo‐Barnes et al., 2020; Gorriz et al., 2021; Jimenez‐Mesa, Ramirez, et al., 2023). This is because it takes advantage of all the samples in the set to fine‐tune the classification approach (resubstitution), adjusting the results a posteriori without bias (upper bounding). This is clearly seen in the PLS component and sample size studies in Figure 8. For example, while the performance using CV was very similar for different PLS values, RUB managed to improve performance when the number of components was small. With the sample size used in this study something similar happened. RUB managed to improve the results with increasing sample size, while CV remained inconsistent for any sample size. The former was expected, since by increasing the set, the classifier's learning should theoretically improve.

The contrast between validation approaches is also seen in Table 2. In this case, by working with a slightly larger number of features, 9, the upper bound obtained to apply in RUB was large, and therefore better results were obtained by applying K‐fold. Conversely, when the number of features was 4 (PLS column), the best performance was again achieved by using RUB, irrespective of the upper bound applied, see Figure 9. In this figure, when using K‐fold, the generalisation capacity of the algorithm was lost. RUB managed to maintain results close to those obtained with the most relevant features in the first columns. Consistently better results were obtained using only the most relevant features compared to dimensionality reduction techniques. This is because in the feature extraction process, all analysed regions were included.

The results in Table 2 also indicate better results when using the features selected by parametric rather than non‐parametric methods. The difference in accuracy is <4%, so both methods were feasible to use. This suggests that in the absence of normal distributions or with a reduced sample size, non‐parametric techniques are a tempting option. However, Figure 7 shows how both methods report relevant features.

Future study will expand the analysis to include the interaction between features, as well as comparisons between sulcal and gyral morphological features. For this purpose, further processing tools will be tested, such as calcSulc and Freesurfer with a multidisciplinary working group, in order to be able to analyse in detail all the results obtained. It would also be useful to expand the database to be able to verify the results obtained on an independent dataset. It would even be highly interesting if such an extension could include databases from different regions in order to be able to detect the environmental impact on schizophrenia. Moreover, more specific studies could be conducted, such as the identification of patterns in those who suffer from hallucinations.

## 6 CONCLUSION

In this study, we evaluated the potential of several ML and DL techniques combined with sulcal features to undertake a novel case–control classification task with schizophrenia patients. Sulcal features were obtained automatically through BrainVISA and encompass the entire cerebral cortex. These features were analysed using techniques of feature selection and extraction, considering parametric and non‐parametric approaches. Then, different classification scenarios were implemented to evaluate the relevance of the features and the performance of the validation methods (resubstitution with upper bound correction and K‐fold cross‐validation) given the circumstances of the study, both in terms of number of samples and features. Explainable artificial intelligence techniques were also applied to detect regions of interest in schizophrenia and to compare their findings with those obtained from feature selection techniques. The performance achieved reflects potentially interesting features that have not previously been reported in terms of length and/or depth, such as the collateral fissure or the superior postcentral intraparietal superior sulcus. Moreover, expected results are obtained in temporal or precentral areas. This study makes manifest the issues involved with classification tasks using novel features obtained from small sample‐size datasets. The techniques described give a roadmap for how researchers might approach a similar problem, and indicates how dimensionality reduction (feature extraction) techniques and validation methods such as upper‐bounding resubstitution help to mitigate the inherent difficulties.

## FUNDING INFORMATION

CIN/AEI/10.13039/501100011033 and by FSE+ (PID2022‐137451OB‐I00, PID2022‐137629OA‐I00); Ministerio de Universidades (FPU18/04902); Medical Research Council (MR/W020025/1); NIHR Cambridge Biomedical Research Centre (NIHR203312).

## CONFLICT OF INTEREST STATEMENT

The authors declare no conflicts of interest.

## Supporting information


**DATA S1.** Supporting Information

## Data Availability

The data that support the findings of this study are available on request from the corresponding author. The data are not publicly available due to privacy or ethical restrictions.

## References

[hbm26555-bib-0001] Andreasen, N. C. , Harris, G. , Cizadlo, T. , Arndt, S. , O'Leary, D. S. , Swayze, V. , & Flaum, M. (1994). Techniques for measuring sulcal/gyral patterns in the brain as visualized through magnetic resonance scanning: BRAINPLOT and BRAINMAP. Proceedings of the National Academy of Sciences, 91(1), 93–97. 10.1073/pnas.91.1.93 PMC42892 8278413

[hbm26555-bib-0002] Arco, J. E. , Ortiz, A. , Castillo‐Barnes, D. , Górriz, J. M. , & Ramírez, J. (2022). Quantifying inter‐hemispheric differences in parkinson's disease using siamese networks. In Artificial intelligence in neuroscience: Affective analysis and health applications: 9th International work‐conference on the interplay between natural and artificial computation, IWINAC 2022, Puerto de la Cruz, Tenerife, Spain, May 31–June 3, 2022, proceedings, part I (pp. 156–165). Springer.

[hbm26555-bib-0003] Auzias, G. , Brun, L. , Deruelle, C. , & Coulon, O. (2015). Deep sulcal landmarks: Algorithmic and conceptual improvements in the definition and extraction of sulcal pits. NeuroImage, 111, 12–25. 25676916 10.1016/j.neuroimage.2015.02.008

[hbm26555-bib-0004] Beeston, C. J. , & Taylor, C. J. (2000). Automatic landmarking of cortical sulci. In Medical image computing and computer‐assisted intervention: MICCAI 2000 (pp. 125–133). Springer.

[hbm26555-bib-0005] Behnke, K. J. , Rettmann, M. E. , Pham, D. L. , Shen, D. , Resnick, S. M. , Davatzikos, C. , & Prince, J. L. (2003). Automatic classification of sulcal regions of the human brain cortex using pattern recognition. In M. Sonka & J. M. Fitzpatrick (Eds.), SPIE Proceedings. SPIE.

[hbm26555-bib-0006] Borne, L. , Rivière, D. , Mancip, M. , & Mangin, J.‐F. (2020). Automatic labeling of cortical sulci using patch‐ or CNN‐based segmentation techniques combined with bottom‐up geometric constraints. Medical Image Analysis, 62, 101651. 32163879 10.1016/j.media.2020.101651

[hbm26555-bib-0007] Cachia, A. , Borst, G. , Jardri, R. , Raznahan, A. , Murray, G. K. , Mangin, J.‐F. , & Plaze, M. (2021). Towards deciphering the fetal foundation of normal cognition and cognitive symptoms from sulcation of the cortex. Frontiers in Neuroanatomy, 15, 712862. 34650408 10.3389/fnana.2021.712862 PMC8505772

[hbm26555-bib-0008] Cachia, A. , Paillère‐Martinot, M.‐L. , Galinowski, A. , Januel, D. , de Beaurepaire, R. , Bellivier, F. , Artiges, E. , Andoh, J. , Bartrés‐Faz, D. , Duchesnay, E. , Rivière, D. , Plaze, M. , Mangin, J.‐F. , & Martinot, J.‐L. (2008). Cortical folding abnormalities in schizophrenia patients with resistant auditory hallucinations. NeuroImage, 39(3), 927–935. 17988891 10.1016/j.neuroimage.2007.08.049

[hbm26555-bib-0009] Campero, A. , Ajler, P. , Emmerich, J. , Goldschmidt, E. , Martins, C. , & Rhoton, A. (2014). Brain sulci and gyri: A practical anatomical review. Journal of Clinical Neuroscience, 21(12), 2219–2225. 25092274 10.1016/j.jocn.2014.02.024

[hbm26555-bib-0010] Castillo‐Barnes, D. , Li, S. , Ramírez, J. , Salas‐Gonzalez, D. , Martinez‐Murcia, F. J. , Illan, I. A. , Segovia, F. , Ortiz, A. , Cruchaga, C. , Farlow, M. R. , Xiong, C. , Graff‐Radford, N. R. , Schofield, P. R. , Masters, C. L. , Salloway, S. , Jucker, M. , Mori, H. , Levin, J. , & Juan, M. (2020). Gorriz, and dominantly inherited Alzheimer network (DIAN). Autosomal dominantly inherited alzheimer disease: Analysis of genetic subgroups by machine learning. Information Fusion, 58, 153–167. 32284705 10.1016/j.inffus.2020.01.001 PMC7153760

[hbm26555-bib-0011] Collantoni, E. , Madan, C. R. , Meregalli, V. , Meneguzzo, P. , Marzola, E. , Panero, M. , D'Agata, F. , Abbate‐Daga, G. , Tenconi, E. , Manara, R. , & Favaro, A. (2021). Sulcal characteristics patterns and gyrification gradient at different stages of anorexia nervosa: A structural MRI evaluation. Psychiatry Research: Neuroimaging, 316, 111350. 34384959 10.1016/j.pscychresns.2021.111350

[hbm26555-bib-0012] Csernansky, J. G. , Gillespie, S. K. , Dierker, D. L. , Anticevic, A. , Wang, L. , Barch, D. M. , & Van Essen, D. C. (2008). Symmetric abnormalities in sulcal patterning in schizophrenia. NeuroImage, 43(3), 440–446. 18707008 10.1016/j.neuroimage.2008.07.034 PMC3011823

[hbm26555-bib-0013] Eklund, A. , Nichols, T. E. , & Knutsson, H. (2016). Cluster failure: Why fmri inferences for spatial extent have inflated false‐positive rates. Proceedings of the National Academy of Sciences, 113(28), 7900–7905. 10.1073/pnas.1602413113 PMC4948312 27357684

[hbm26555-bib-0014] Fay, M. P. , & Proschan, M. A. (2010). Wilcoxon–Mann–Whitney or t‐test? On assumptions for hypothesis tests and multiple interpretations of decision rules. Statistics Surveys, 4, 1–39. 20414472 10.1214/09-SS051 PMC2857732

[hbm26555-bib-0015] Garrison, J. R. , Fernyhough, C. , McCarthy‐Jones, S. , Haggard, M. , Australian Schizophrenia Research Bank , & Simons, J. S. (2015). Paracingulate sulcus morphology is associated with hallucinations in the human brain. Nature Communications, 6(1), 8956. 10.1038/ncomms9956 PMC4660352 26573408

[hbm26555-bib-0016] Geffroy, D. , Rivière, D. , Denghien, I. , Souedet, N. , Laguitton, S. , & Cointepas, Y. (2011). Brainvisa: A complete software platform for neuroimaging. In Python in neuroscience Workshop, Paris.

[hbm26555-bib-0017] Gorriz, J. M. , Jimenez‐Mesa, C. , Romero‐Garcia, R. , Segovia, F. , Ramirez, J. , Castillo‐Barnes, D. , Martinez‐Murcia, F. J. , Ortiz, A. , Salas‐Gonzalez, D. , Illan, I. A. , Puntonet, C. G. , Lopez‐Garcia, D. , Gomez‐Rio, M. , & Suckling, J. (2021). Statistical agnostic mapping: A framework in neuroimaging based on concentration inequalities. Information Fusion, 66, 198–212.

[hbm26555-bib-0018] Gorriz, J. M. , Ramirez, J. , Suckling, J. , Illan, I. A. , Andres Ortiz, F. J. , Martinez‐Murcia, F. S. , Salas‐Gonzalez, D. , & Wang, S. (2017). Case‐based statistical learning: A non‐parametric implementation with a conditional‐error rate SVM. IEEE Access, 5, 11468–11478.

[hbm26555-bib-0019] Górriz, J. M. , Ramírez, J. , Ortz, A. , Martínez‐Murcia, F. J. , Segovia, F. , Suckling, J. , Leming, M. , Zhang, Y.‐D. , Álvarez‐Sánchez, J. R. , Bologna, G. , Bonomini, P. , Casado, F. E. , Charte, D. , Charte, F. , Contreras, R. , Cuesta‐Infante, A. , Duro, R. J. , Fernández‐Caballero, A. , Fernández‐Jover, E. , … Ferrández, J. M. (2020). Artificial intelligence within the interplay between natural and artificial computation: Advances in data science, trends and applications. Neurocomputing, 410, 237–270.

[hbm26555-bib-0020] Górriz, J. M. , Ramirez, J. , & Suckling, J. (2019). On the computation of distribution‐free performance bounds: Application to small sample sizes in neuroimaging. Pattern Recognition, 93, 1–13.

[hbm26555-bib-0021] Graña, M. , Termenon, M. , Savio, A. , Gonzalez‐Pinto, A. , Echeveste, J. , Pérez, J. M. , & Besga, A. (2011). Computer aided diagnosis system for alzheimer disease using brain diffusion tensor imaging features selected by pearson's correlation. Neuroscience Letters, 502(3), 225–229. 21839143 10.1016/j.neulet.2011.07.049

[hbm26555-bib-0022] Gunning, D. , Stefik, M. , Choi, J. , Miller, T. , Stumpf, S. , & Yang, G.‐Z. (2019). XAI: Explainable artificial intelligence. Science Robotics, 4(37), eaay7120. 10.1126/scirobotics.aay7120 33137719

[hbm26555-bib-0023] Hajian‐Tilaki, K. (2013). Receiver operating characteristic (roc) curve analysis for medical diagnostic test evaluation. Caspian Journal of Internal Medicine, 4(2), 627–635. 24009950 PMC3755824

[hbm26555-bib-0024] Hope Weissler, E. , Naumann, T. , Andersson, T. , Ranganath, R. , Elemento, O. , Luo, Y. , Freitag, D. F. , Benoit, J. , Hughes, M. C. , Khan, F. , Slater, P. , Shameer, K. , Roe, M. , Hutchison, E. , Kollins, S. H. , Broedl, U. , Meng, Z. , Wong, J. L. , Curtis, L. , & Ghassemi, E. H. M. (2021). The role of machine learning in clinical research: Transforming the future of evidence generation. Trials, 22(1), 1–15. 34399832 10.1186/s13063-021-05489-x PMC8365941

[hbm26555-bib-0025] Ioannidis, J. P. A. (2005). Why most published research findings are false. PLoS Medicine, 2(8), e124. 16060722 10.1371/journal.pmed.0020124 PMC1182327

[hbm26555-bib-0026] Janssen, J. , Alloza, C. , Daz‐Caneja, C. M. , Santonja, J. , Pina‐Camacho, L. , Gordaliza, P. M. , Fernández‐Pena, A. , Lois, N. G. , Buimer, E. E. L. , van Haren, N. E. M. , Cahn, W. , Vieta, E. , Castro‐Fornieles, J. , Bernardo, M. , Arango, C. , Kahn, R. S. , Hulshoff, H. E. , & Schnack, H. G. (2022). Longitudinal allometry of sulcal morphology in health and schizophrenia. The Journal of Neuroscience, 42(18), 3704–3715. 35318286 10.1523/JNEUROSCI.0606-21.2022 PMC9087719

[hbm26555-bib-0027] Javier Ramírez, J. M. , Górriz, D. S.‐G. , Romero, A. , López, M. , Álvarez, I. , & Gómez‐Ro, M. (2013). Computer‐aided diagnosis of alzheimer's type dementia combining support vector machines and discriminant set of features. Information Sciences, 237, 59–72.

[hbm26555-bib-0028] Jimenez‐Mesa, C. , Arco, J. E. , Valenti‐Soler, M. , Frades‐Payo, B. , Zea‐Sevilla, M. A. , Ortiz, A. , Avila‐Villanueva, M. , Castillo‐Barnes, D. , Ramirez, J. , Del Ser‐Quijano, T. , Carnero‐Pardo, C. , & Gorriz, J. M. (2023). Using explainable artificial intelligence in the clock drawing test to reveal the cognitive impairment pattern. International Journal of Neural Systems, 33, 2350015. 36799660 10.1142/S0129065723500156

[hbm26555-bib-0029] Jimenez‐Mesa, C. , Illan, I. A. , Martin‐Martin, A. , Castillo‐Barnes, D. , Martinez‐Murcia, F. J. , Ramirez, J. , & Gorriz, J. M. (2020). Optimized one vs one approach in multiclass classification for early alzheimer's disease and mild cognitive impairment diagnosis. IEEE Access, 8, 96981–96993.

[hbm26555-bib-0030] Jimenez‐Mesa, C. , Ramirez, J. , Suckling, J. , Vöglein, J. , Levin, J. , & Gorriz, J. M. (2023). A non‐parametric statistical inference framework for deep learning in current neuroimaging. Information Fusion, 91, 598–611.

[hbm26555-bib-0031] Jin, K. , Zhang, T. , Shaw, M. , Sachdev, P. , & Cherbuin, N. (2018). Relationship between sulcal characteristics and brain aging. Frontiers in Aging Neuroscience, 10, 339. 10.3389/fnagi.2018.00339 PMC6240579 30483112

[hbm26555-bib-0032] John, J. P. , Wang, L. , Moffitt, A. J. , Singh, H. K. , Gado, M. H. , & Csernansky, J. G. (2006). Inter‐rater reliability of manual segmentation of the superior, inferior and middle frontal gyri. Psychiatry Research: Neuroimaging, 148(2–3), 151–163. 10.1016/j.pscychresns.2006.05.006 17088050

[hbm26555-bib-0033] Kim, T. K. (2015). T test as a parametric statistic. Korean Journal of Anesthesiology, 68(6), 540–546. 26634076 10.4097/kjae.2015.68.6.540 PMC4667138

[hbm26555-bib-0034] Kohavi, R. (1995). A study of cross‐validation and bootstrap for accuracy estimation and model selection. In International Joint Conference on Artificial Intelligence (IJCAI) (Vol. 14, pp. 1137–1145). Montreal.

[hbm26555-bib-0035] Leming, M. , Górriz, J. M. , & Suckling, J. (2020). Ensemble deep learning on large, mixed‐site fMRI datasets in autism and other tasks. International Journal of Neural Systems, 30(7), 2050012. 32308082 10.1142/S0129065720500124

[hbm26555-bib-0036] Leming, M. J. , Baron‐Cohen, S. , & Suckling, J. (2021). Single‐participant structural similarity matrices lead to greater accuracy in classification of participants than function in autism in MRI. Molecular Autism, 12(1), 34. 33971956 10.1186/s13229-021-00439-5 PMC8112019

[hbm26555-bib-0037] Li, Z. , Yan, C. , Lv, Q.‐y. , Yi, Z.‐h. , Zhang, J.‐y. , Wang, J.‐h. , Lui, S. S. Y. , Yi‐feng, X. , Cheung, E. F. C. , Gur, R. E. , Gur, R. C. , & Chan, R. C. K. (2018). Striatal dysfunction in patients with schizophrenia and their unaffected first‐degree relatives. Schizophrenia Research, 195, 215–221. 28867519 10.1016/j.schres.2017.08.043

[hbm26555-bib-0038] Liu, N. , Xiao, Y. , Zhang, W. , Tang, B. , Zeng, J. , Na, H. , Chandan, S. , Gong, Q. , & Lui, S. (2020). Characteristics of gray matter alterations in never‐treated and treated chronic schizophrenia patients. Translational Psychiatry, 10(1), 136. 32398765 10.1038/s41398-020-0828-4 PMC7217843

[hbm26555-bib-0039] Lombardi, A. , Diacono, D. , Amoroso, N. , Biecek, P. , Monaco, A. , Bellantuono, L. , Pantaleo, E. , Logroscino, G. , de Blasi, R. , Tangaro, S. , & Bellotti, R. (2022). A robust framework to investigate the reliability and stability of explainable artificial intelligence markers of mild cognitive impairment and alzheimer's disease. Brain Informatics, 9(1), 17. 35882684 10.1186/s40708-022-00165-5 PMC9325942

[hbm26555-bib-0040] Lombardi, A. , Diacono, D. , Amoroso, N. , Monaco, A. , João Manuel, R. S. , Tavares, R. B. , & Tangaro, S. (2021). Explainable deep learning for personalized age prediction with brain morphology. Frontiers in Neuroscience, 15, 578. 10.3389/fnins.2021.674055 PMC8192966 34122000

[hbm26555-bib-0041] Louis Collins, D. , Neelin, P. , Peters, T. M. , & Evans, A. C. (1994). Automatic 3d intersubject registration of mr volumetric data in standardized talairach space. Journal of Computer Assisted Tomography, 18(2), 192–205. 8126267

[hbm26555-bib-0042] Lundberg, S. M. , & Lee, S.‐I. (2017). A unified approach to interpreting model predictions. In I. Guyon , U. V. Luxburg , S. Bengio , H. Wallach , R. Fergus , S. Vishwanathan , & R. Garnett (Eds.), Advances in neural information processing systems 30 (pp. 4765–4774). Curran Associates, Inc.

[hbm26555-bib-0043] Madan, C. R. (2019). Robust estimation of sulcal morphology. Brain Informatics, 6(1), 5. 31187294 10.1186/s40708-019-0098-1 PMC6560124

[hbm26555-bib-0044] Mandrekar, J. N. (2010). Receiver operating characteristic curve in diagnostic test assessment. Journal of Thoracic Oncology, 5(9), 1315–1316. 20736804 10.1097/JTO.0b013e3181ec173d

[hbm26555-bib-0045] Mann, H. B. , & Whitney, D. R. (1947). On a test of whether one of two random variables is stochastically larger than the other. The Annals of Mathematical Statistics, 18(1), 50–60.

[hbm26555-bib-0046] Martinez‐Murcia, F. J. , Górriz, J. M. , Ramírez, J. , & Ortiz, A. (2018). Convolutional neural networks for neuroimaging in parkinson's disease: Is preprocessing needed? International Journal of Neural Systems, 28(10), 1850035. 30215285 10.1142/S0129065718500351

[hbm26555-bib-0047] Mateos, M. J. , Gastelum‐Strozzi, A. , Barrios, F. A. , Bribiesca, E. , Alcauter, S. , & Marquez‐Flores, J. A. (2020). A novel voxel‐based method to estimate cortical sulci width and its application to compare patients with alzheimer's disease to controls. NeuroImage, 207, 116343. 31734431 10.1016/j.neuroimage.2019.116343

[hbm26555-bib-0048] McAllester, D. (2013). A pac‐bayesian tutorial with a dropout bound. arXiv Preprint, arXiv:1307.2118.

[hbm26555-bib-0049] McAlonan, G. M. (2004). Mapping the brain in autism. A voxel‐based MRI study of volumetric differences and intercorrelations in autism. Brain, 128(2), 268–276. 15548557 10.1093/brain/awh332

[hbm26555-bib-0050] Mellerio, C. , Lapointe, M.‐N. , Roca, P. , Charron, S. , Legrand, L. , Meder, J.‐F. , Oppenheim, C. , & Cachia, A. (2016). Identification of reliable sulcal patterns of the human rolandic region. Frontiers in Human Neuroscience, 10, 410. 10.3389/fnhum.2016.00410 PMC4987365 27582700

[hbm26555-bib-0051] Mikhael, S. , Hoogendoorn, C. , Valdes‐Hernandez, M. , & Pernet, C. (2018). A critical analysis of neuroanatomical software protocols reveals clinically relevant differences in parcellation schemes. NeuroImage, 170, 348–364. 28279814 10.1016/j.neuroimage.2017.02.082

[hbm26555-bib-0052] Murphy, S. , Mohr, B. , Fushimi, Y. , Yamagata, H. , & Poole, I. (2014). Fast, simple, accurate multi‐atlas segmentation of the brain. In Biomedical Image Registration: 6th International Workshop, WBIR 2014, London, UK, July 7–8, 2014. Proceedings 6 (pp. 1–10). Springer.

[hbm26555-bib-0053] Nesvåg, R. , Schaer, M. , Haukvik, U. K. , Westlye, L. T. , Rimol, L. M. , Lange, E. H. , Hartberg, C. B. , Ottet, M.‐C. , Melle, I. , Andreassen, O. A. , Jönsson, E. G. , Agartz, I. , & Eliez, S. (2014). Reduced brain cortical folding in schizophrenia revealed in two independent samples. Schizophrenia Research, 152(2–3), 333–338. 24365403 10.1016/j.schres.2013.11.032

[hbm26555-bib-0054] Orru, G. , Pettersson‐Yeo, W. , Marquand, A. F. , Sartori, G. , & Mechelli, A. (2012). Using support vector machine to identify imaging biomarkers of neurological and psychiatric disease: A critical review. Neuroscience & Biobehavioral Reviews, 36(4), 1140–1152. 22305994 10.1016/j.neubiorev.2012.01.004

[hbm26555-bib-0055] Ortiz, A. , Munilla, J. , Górriz, J. M. , & Ramírez, J. (2016). Ensembles of deep learning architectures for the early diagnosis of the alzheimer's disease. International Journal of Neural Systems, 26(7), 1650025. 27478060 10.1142/S0129065716500258

[hbm26555-bib-0056] Palaniyappan, L. , Hodgson, O. , Balain, V. , Iwabuchi, S. , Gowland, P. , & Liddle, P. (2018). Structural covariance and cortical reorganisation in schizophrenia: A MRI‐based morphometric study. Psychological Medicine, 49(3), 412–420. 29729682 10.1017/S0033291718001010

[hbm26555-bib-0057] Palaniyappan, L. , Park, B. , Balain, V. , Dangi, R. , & Liddle, P. (2014). Abnormalities in structural covariance of cortical gyrification in schizophrenia. Brain Structure and Function, 220(4), 2059–2071. 24771247 10.1007/s00429-014-0772-2 PMC4481329

[hbm26555-bib-0058] Panagiotakos, D. B. (2008). The value of *p*‐value in biomedical research. The Open Cardiovascular Medicine Journal, 2, 97–99. 19430522 10.2174/1874192400802010097 PMC2627527

[hbm26555-bib-0059] Perrot, M. , Rivière, D. , & Mangin, J.‐F. (2011). Cortical sulci recognition and spatial normalization. Medical Image Analysis, 15(4), 529–550. 21441062 10.1016/j.media.2011.02.008

[hbm26555-bib-0060] Pizzagalli, F. , Auzias, G. , Kochunov, P. , Faskowitz, J. I. , Thompson, P. M. , & Jahanshad, N. (2017). The core genetic network underlying sulcal morphometry. In E. Romero , N. Lepore , J. Brieva , & I. Larrabide (Eds.), SPIE Proceedings. SPIE.

[hbm26555-bib-0061] Plocharski, M. , & Østergaard, L. R. (2016). Extraction of sulcal medial surface and classification of alzheimer's disease using sulcal features. Computer Methods and Programs in Biomedicine, 133, 35–44. 27393798 10.1016/j.cmpb.2016.05.009

[hbm26555-bib-0062] Ribeiro, M. T. , Singh, S. , & Guestrin, C. (2016). “Why should I trust you?”: Explaining the predictions of any classifier. In Proceedings of the 22nd ACM SIGKDD International Conference on Knowledge Discovery and Data Mining, San Francisco, CA, USA, August 13–17, 2016 (pp. 1135–1144). ACM.

[hbm26555-bib-0063] Ribolsi, M. , Daskalakis, Z. J. , Siracusano, A. , & Koch, G. (2014). Abnormal asymmetry of brain connectivity in schizophrenia. Frontiers in Human Neuroscience, 8, 1010. 10.3389/fnhum.2014.01010 PMC4273663 25566030

[hbm26555-bib-0064] Rollins, C. P. E. , Garrison, J. R. , Arribas, M. , Seyedsalehi, A. , Li, Z. , Chan, R. C. K. , Yang, J. , Wang, D. , Liò, P. , Yan, C. , Yi, Z. H. , Cachia, A. , Upthegrove, R. , Deakin, B. , Simons, J. S. , Murray, G. K. , & Suckling, J. (2020). Evidence in cortical folding patterns for prenatal predispositions to hallucinations in schizophrenia. Translational Psychiatry, 10(1), 387. 10.1038/s41398-020-01075-y PMC7648757 33159044

[hbm26555-bib-0065] Sallet, P. C. , Elkis, H. , Alves, T. M. , Oliveira, J. R. , Sassi, E. , de Castro, C. C. , Busatto, G. F. , & Gattaz, W. F. (2003). Reduced cortical folding in schizophrenia: An MRI morphometric study. American Journal of Psychiatry, 160(9), 1606–1613. 12944334 10.1176/appi.ajp.160.9.1606

[hbm26555-bib-0066] Schaer, M. , Cuadra, M. B. , Tamarit, L. , Lazeyras, F. , Eliez, S. , & Thiran, J.‐P. (2008). A surface‐based approach to quantify local cortical gyrification. IEEE Transactions on Medical Imaging, 27(2), 161–170. 18334438 10.1109/TMI.2007.903576

[hbm26555-bib-0067] Scheda, R. , & Diciotti, S. (2022). Explanations of machine learning models in repeated nested cross‐validation: An application in age prediction using brain complexity features. Applied Sciences, 12(13), 6681.

[hbm26555-bib-0068] Schölkopf, B. , & Smola, A. J. (2002). Learning with kernels: Support vector machines, regularization, optimization, and beyond. MIT Press.

[hbm26555-bib-0069] Shapiro, S. S. , & Wilk, M. B. (1965). An analysis of variance test for normality (complete samples). Biometrika, 52(3–4), 591–611.

[hbm26555-bib-0070] Shapley, L. S. (1953). A value for n‐person games. Annals of Mathematical Studies, 28, 307–317.

[hbm26555-bib-0071] Shen, X. , Liu, T. , Tao, D. , Fan, Y. , Zhang, J. , Li, S. , Jiang, J. , Zhu, W. , Wang, Y. , Wang, Y. , Brodaty, H. , Sachdev, P. , & Wen, W. (2018). Variation in longitudinal trajectories of cortical sulci in normal elderly. NeuroImage, 166, 1–9. 29080713 10.1016/j.neuroimage.2017.10.010

[hbm26555-bib-0072] Shu Lih, O. , Yuki Hagiwara, U. , Raghavendra, R. Y. , Arunkumar, N. , Murugappan, M. , & Rajendra Acharya, U. (2018). A deep learning approach for parkinson's disease diagnosis from EEG signals. Neural Computing and Applications, 32(15), 10927–10933.

[hbm26555-bib-0073] Talairach, J . (1988). Co‐planar stereotaxic atlas of the human brain. 3‐D proportional system: An approach to cerebral imaging. Thieme.

[hbm26555-bib-0074] van der Velden, B. H. M. , Kuijf, H. J. , Gilhuijs, K. G. A. , & Viergever, M. A. (2022). Explainable artificial intelligence (xai) in deep learning‐based medical image analysis. Medical Image Analysis, 79, 102470. 35576821 10.1016/j.media.2022.102470

[hbm26555-bib-0075] Vapnik, V. N. (1982). Estimation of dependencies based on empirical data. Springer.

[hbm26555-bib-0076] Vapnik, V. , Levin, E. , & Le Cun, Y. (1994). Measuring the vc‐dimension of a learning machine. Neural Computation, 6(5), 851–876.

[hbm26555-bib-0077] Wagner, A. , Ruf, M. , Braus, D. F. , & Schmidt, M. H. (2003). Neuronal activity changes and body image distortion in anorexia nervosa. Neuroreport, 14(17), 2193–2197. 14625446 10.1097/00001756-200312020-00012

[hbm26555-bib-0078] Wang, J. , You, H. , Liu, J.‐F. , Ni, D.‐F. , Zhang, Z.‐X. , & Guan, J. (2011). Association of olfactory bulb volume and olfactory sulcus depth with olfactory function in patients with parkinson disease. American Journal of Neuroradiology, 32(4), 677–681. 21330398 10.3174/ajnr.A2350 PMC7965889

[hbm26555-bib-0079] Welch, B. L. (1947). The generalization of student's problem when several different population varlances are involved. Biometrika, 34(1–2), 28–35. 20287819 10.1093/biomet/34.1-2.28

[hbm26555-bib-0080] Wold, S. , Ruhe, A. , Wold, H. , & Dunn, I. I. I. W. J. (1984). The collinearity problem in linear regression. The partial least squares (PLS) approach to generalized inverses. SIAM Journal on Scientific and Statistical Computing, 5(3), 735–743.

[hbm26555-bib-0081] Wright, I. C. , Ellison, Z. R. , Sharma, T. , Friston, K. J. , Murray, R. M. , & McGuire, P. K. (1999). Mapping of grey matter changes in schizophrenia. Schizophrenia Research, 35(1), 1–14. 9988836 10.1016/s0920-9964(98)00094-2

[hbm26555-bib-0082] Yang, F. , & Kruggel, F. (2008). Automatic segmentation of human brain sulci. Medical Image Analysis, 12(4), 442–451. 18325826 10.1016/j.media.2008.01.003

[hbm26555-bib-0083] Yang, J. , Wang, D. , Rollins, C. , Leming, M. , Liò, P. , Suckling, J. , Murray, G. , Garrison, J. , & Cachia, A. (2019). Volumetric segmentation and characterisation of the paracingulate sulcus on mri scans. bioRxiv, 859496.

[hbm26555-bib-0084] Yücel, M. , Stuart, G. W. , Maruff, P. , Wood, S. J. , Savage, G. R. , Smith, D. J. , Crowe, S. F. , Copolov, D. L. , Velakoulis, D. , & Pantelis, C. (2002). Paracingulate morphologic differences in males with established schizophrenia: A magnetic resonance imaging morphometric study. Biological Psychiatry, 52(1), 15–23. 12079726 10.1016/s0006-3223(02)01312-4

[hbm26555-bib-0085] Zhang, Y. , Lin, L. , Lin, C.‐P. , Zhou, Y. , Chou, K.‐H. , Lo, C.‐Y. , Tung‐Ping, S. , & Jiang, T. (2012). Abnormal topological organization of structural brain networks in schizophrenia. Schizophrenia Research, 141(2–3), 109–118. 22981811 10.1016/j.schres.2012.08.021

