# Supplementary material for: Machine learning in small sample neuroimaging studies: Novel measures for schizophrenia analysis
Source: Hum Brain Mapp. 2024 Mar 27;45(5):e26555. doi: 10.1002/hbm.26555 (PMC10973702; doi:10.1002/hbm.26555)
Supplement: Supplementary file 1 — DATA S1. Supporting Information [file HBM-45-e26555-s001.pdf]

Supplementary Material

Detailed description of the information obtained with SHAP

A summary plot including the impact on the classification model of the 147 features is illustrated in Figure 1. The order selected was from Frontal lobe to Occipital lobe, which are arranged from from left to right and from top to bottom on the illustration.

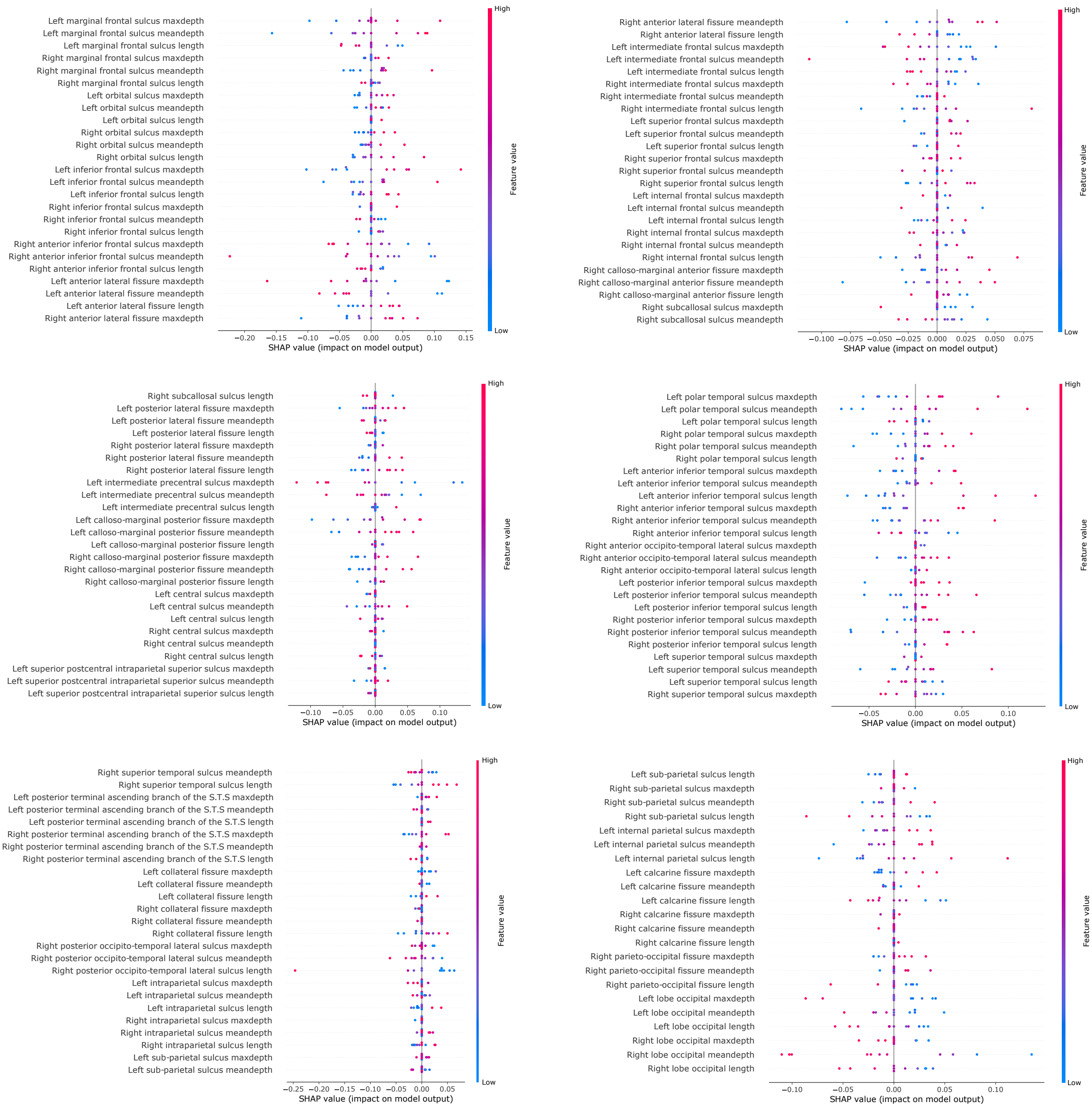

Figure 1. Summary plot of features importance in the classification decision. These features are arranged from Frontal lobe to Occipital lobe (from left to right and from top to bottom). The positive class is SCZ. Each point represents an instance of the test sample. Average and maximum depth are abbreviated as meandepth and maxdepth.
